# Mechanisms of Aphids (*Myzus persicae* (Sulzer)) Response to Insecticides and Drought Stresses on Cabbage (*Brassica rapa* L. ssp. Pekinensis)

**DOI:** 10.3390/plants15020219

**Published:** 2026-01-10

**Authors:** Peter Quandahor, Jong-ho Park, Minju Kim, Keunpyo Lee, Ahrang Kang, Young Ho Koh, Francis Kusi, Mohammed Mujitaba Dawuda, Jerry A. Nboyine, In-hong Jeong

**Affiliations:** 1 Pests and Weeds Control Division, National Institute of Agricultural Sciences, Rural Development Administration, Wanju 55365, Republic of Korea; quandooh@yahoo.com (P.Q.);; 2 Council for Scientific and Industrial Research (CSIR), Savanna Agricultural Research Institute (SARI), Tamale P.O. Box TL 52, Ghana; 3 Toxicity and Risk Assessment Division, National Institute of Agricultural Sciences, Rural Development Administration, Wanju 55365, Republic of Korea; 4 International Technology Cooperation Center, Rural Development Administration, Wanju 55365, Republic of Korea; 5 Ilsong Institute of Life Sciences, Hallym University, Seoul 07247, Republic of Korea; 6 Department of Horticulture, Faculty of Agriculture, University for Development Studies, Tamale P.O Box TL 1882, Ghana

**Keywords:** drought stress, insecticides application, aphid’s performance, water use efficiency

## Abstract

Drought stress and insecticide exposure are two significant environmental factors that can impact the physiology and behaviour of aphids, a major agricultural pest. An understanding of the mechanisms of green peach aphids’ response to insecticides under drought stress is a critical area of research that needs urgent attention. In view of this, we conducted this study to determine the impact of drought and insecticides on the activity of detoxification enzymes in green peach aphid. A 2 × 2 × 3 factorial experiment involving two levels of water treatments (drought and no drought), two levels of aphids infestation (aphids and no aphids), and three levels of pesticides applications (thiacloprid, flonicamid and no pesticide) was conducted. The treatments were arranged in a randomized complete block design with three replications. The results showed that there was a significant (*p* < 0.01) interaction effect of drought × insecticides on the green peach aphid performance under drought or no drought conditions. Generally, the highest aphids host acceptance, survival rate, colonization success, and average daily reproduction under drought and well-watered conditions occurred on flonicamid-treated plants, whereas thiacloprid-treated plants had the least. However, the thiacloprid-treated plants had higher photosynthetic rate, water use efficiency, lower stomatal conductance, and decreased transpiration rate. Moreover, flonicamid treatment increased the accumulation of glutathione–S-transferase, acetylcholinesterase, butyrylcholinesterase, 1-napthyle acetate, and 1-napthyle butyrate activities in aphids, compared to the thiacloprid treatments. The thiacloprid pesticide, which demonstrated higher efficacy against green peach aphid, can be used in areas where green peach aphids and drought stress are major concerns.

## 1. Introduction

Insecticide resistance demonstrates microevolution, with rapid adaptation in response to environmental stressors [1]. The increasing resistance of insect pests to insecticides is a major concern for agriculture, forestry, and public health. Agricultural practices involving multiple active compounds create diverse selective pressures that induce resistance [2]. The green peach aphid (*Myzus persicae* (Sulzer)) originated in the Palearctic region and has spread globally, causing significant damage to various crops [3]. It is a polyphagous aphid that infests more than 50 plant families, causing significant losses in agricultural, horticultural, and fruit crops [4]. It is classified as a major agricultural pest [5], with the potential to develop biotype against insecticide [6].

Insect metabolic detoxification depends greatly on detoxification enzymes such as glutathione-S-transferase (GST), acetylcholinesterase (AChE), butyrylcholinesterase (BuChE), and esterases (1-napthyle acetate and 1-napthyle butyrate). These enzymes are induced by a variety of exogenous and endogenous compounds, allowing insects to respond quickly to environmental stresses such as insecticides [7], higher temperature [8], and elevated CO_2_ levels [9]. Research shows that insecticides have a wide range of impacts on insect defence enzyme systems, with changes in enzyme activity associated with insect mortality or resistance development.

Notably, insects exposed to imidacloprid showed an increase in GST and AChE activities [10,11]. In some insect species, sub-lethal concentrations of insecticides such as imidacloprid, diflubenzuron, and abamectin can stimulate carboxylesterase (CarE) and GST activity [12]. Li et al. [13] found that treating western flower thrips (*Frankliniella occidentalis*) with a 25% lethal concentration (LC_25_) dose of spinetoram significantly increased CarE, GST, and cytochrome P450 (CYP450) activity while decreasing AChE activity. Detoxification enzymes are critical for insect survival in adverse conditions [14,15]. The increased enzyme activity suggests that they play a role in mitigating insecticide stress, which modulates insecticide susceptibility.

Chinese cabbage (*Brassica rapa* L. ssp. pekinensis) is generally cultivated during autumn for kimjang due to its preference for cool climates [16]; nevertheless, advances in cultivation technology have enabled the development of summer and winter varieties, allowing for a year-round production. However, drought conditions significantly impede Chinese cabbage growth, resulting in physiological disorders, pest infestations, and lower yields [17]. Drought stress has a significant impact on Chinese cabbage by reducing photosynthesis, transpiration, water use efficiency, stomatal conductance, and plant biomass, resulting in reduced yield and quality [17,18]. Under drought conditions, Chinese cabbage becomes more susceptible to the green peach aphid, whose population increases rapidly due to the accumulation of primary metabolites such as amino acids, sugars, and nitrogen-containing compounds in stressed plants. Currently, chemical pesticides are the most effective management strategy, but their broad-spectrum use has resulted in the development of resistance in target pests [19].

Drought stress and insecticide exposure are two significant environmental factors that can impact the physiology and behaviour of green peach aphids, a major agricultural pest. However, the interactive effects of drought and insecticides on the detoxification mechanisms of these aphids are not well understood. This knowledge gap hinders the development of effective and sustainable pest management strategies, particularly in the context of climate change, which is expected to increase the frequency and severity of drought events. Therefore, this study was aimed at determining the impact of drought and insecticides on the activity of detoxification enzymes in green peach aphid, providing valuable insights for the development of novel pest control approaches.

## 2. Results

### 2.1. Aphids’ Performance

**Figure 1:**
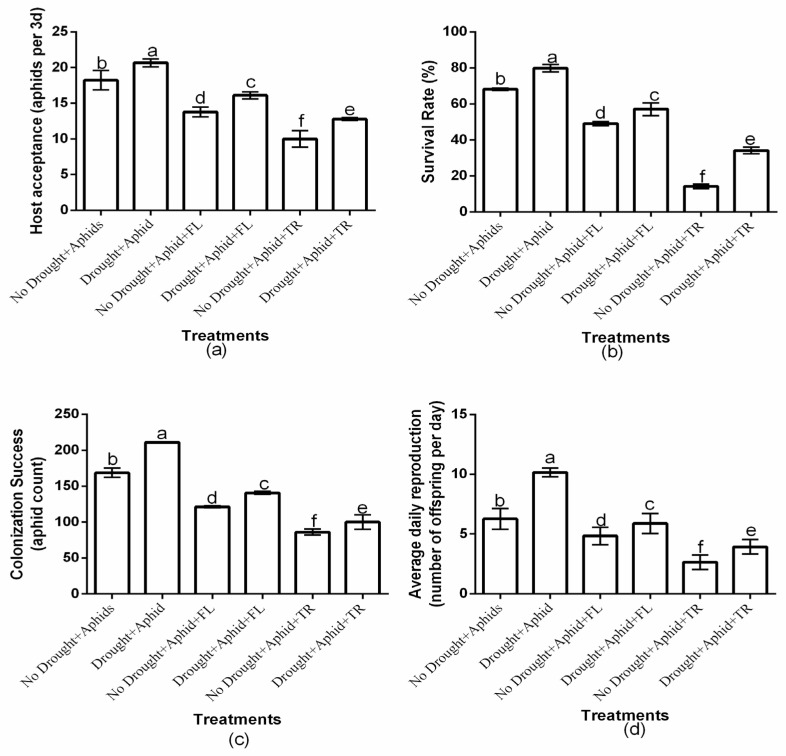
Effects of insecticides on aphid host acceptance (**a**), survival rate (**b**), colonization success (**c**), and daily average daily reproduction (**d**) under drought and well-watered conditions, with or without peach aphids’ infestation. Values are presented as mean ± standard deviation of three independent replicates. Different lowercase letters indicate means that are significantly different according to the LSD test (*p* < 0.05). Drought + FL, Drought + TR, No Drought + FL, and No Drought + TR (FL = flonicamid; TR = thiacloprid).

The study showed a significant (*p* < 0.01) drought × insecticide interaction effect on aphid host acceptance, survival rate, colonization success, and average daily reproduction (Figure 1a–d). Insecticides application had a significant effect on host acceptance (df = 2, F = 35.383, *p* < 0.01), survival rate (df = 2, F = 845.226, *p* < 0.01), colonization success (df = 2, F = 269.825, *p* < 0.01), and average daily reproduction (df = 2, F = 26.943, *p* < 0.01). Moreover, drought condition significantly affected host acceptance (df = 1, F = 51.557, *p* < 0.01), survival rate (df = 1, F = 491.853, *p* < 0.01), colonization success (df = 1, F = 439.610, *p* < 0.01), and average daily reproduction (df = 1, F = 54.886, *p* < 0.01). Notably, under drought and well-watered conditions, flonicamid-treated plants consistently exhibited the highest aphid host acceptance (16.1 ± 0.50 and 13.8 ± 0.69, respectively), survival rates (57.1% ± 3.53 and 49.1% ± 1.07, respectively), colonization success (140.7 ± 2.08 and 121.4 ± 1.34, respectively), and average daily reproduction (5.9 ± 0.84 and 4.9 ± 0.71, respectively). In contrast, thiacloprid-treated plants showed the lowest values for these parameters host acceptance (12.8 ± 0.19 and 10 ± 1.15, respectively), survival rate (34.1% ± 1.83 and 14.2% ± 1.26, respectively), colonization success (100.1 ± 10.07 and 86.2 ± 4.00, respectively), and average daily reproduction (3.9 ± 0.611 and 2.6 ± 0.601, respectively) under drought and well-watered conditions. These findings indicate that thiacloprid was more effective in reducing aphid performance, regardless of drought or well-watered conditions (Figure 1a–d).

### 2.2. Interaction Effect of Drought and Insecticides on Aphid Water Content and Aphid Tolerance Index

**Figure 2:**
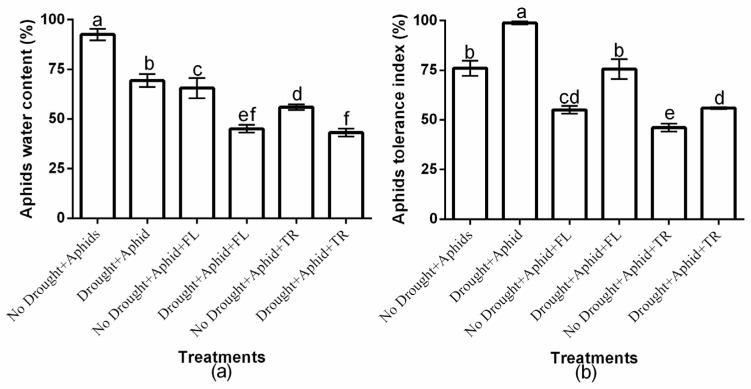
Effect of insecticides on (**a**) aphids water content and (**b**) aphids tolerance index under well-watered or drought conditions. Data represent the mean ± standard deviation of three replicates. Different lowercase letters indicate means that are significantly different according to the LSD test (*p* < 0.05). Drought + FL, Drought + TR, No Drought + FL, and No Drought + TR (FL = flonicamid; TR = thiacloprid).

This study showed significant (*p* < 0.01) drought × insecticide interaction effects on aphid water content and tolerance index (Figure 2a,b). Insecticides application had a significant effect on aphid water content (df = 2, F = 192.591, *p* < 0.01) and tolerance index (df = 2, F = 211.021, *p* < 0.01). Moreover, drought condition significantly affected aphid water content (df = 1, F = 183.583, *p* < 0.01) and tolerance index (df = 1, F = 133.402, *p* < 0.01). Aphid water content was higher on well-watered plants compared to drought-stressed plants. Notably, flonicamid-treated plants exhibited the highest aphid water content under well-watered conditions (65.5% ± 5.03), whereas thiacloprid-treated plants had the least (55.9% ± 1.41). However, difference was not observed under drought stress (Figure 2a). Also, the aphid tolerance index was highest on flonicamid-treated plants under both drought (75.5% ± 5.03) and well-watered conditions (55% ± 1.95), and lowest on thiacloprid-treated plants (55.9% ± 0.41 and 46.1% ± 2.00, respectively) (Figure 2b). Thiacloprid treatments consistently resulted in the lowest aphid water content and tolerance index under both water regimes, suggesting that green peach aphids exhibited greater resilience to flonicamid compared to thiacloprid (Figure 2a,b).

### 2.3. Aphid’s Detoxification Enzymes Activities

**Figure 3:**
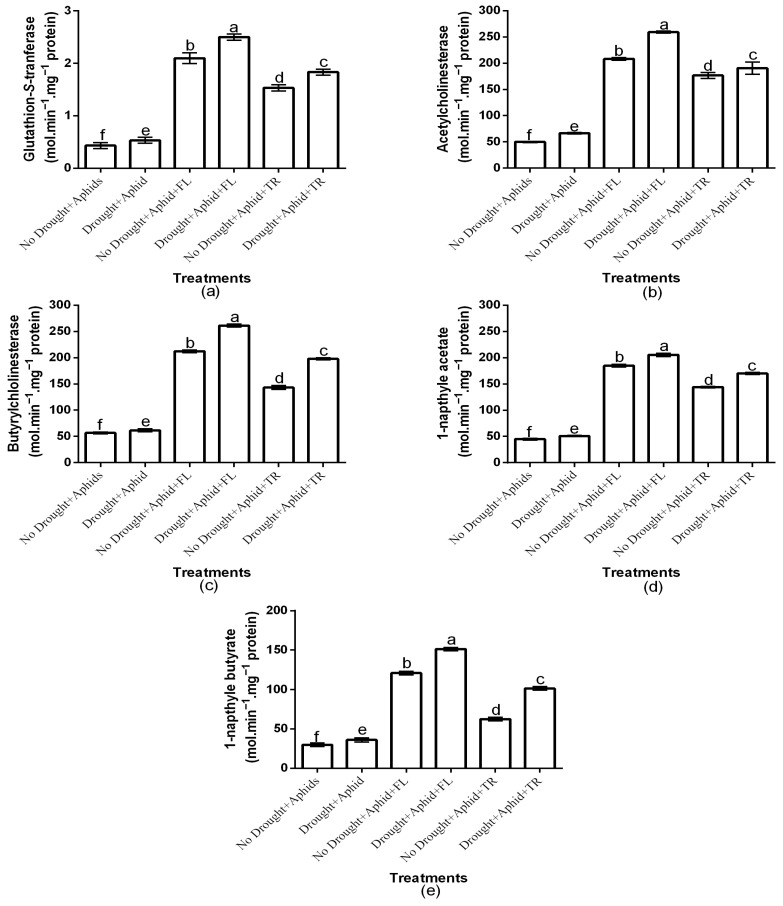
Effects of insecticides on GST (**a**), AChE (**b**), BuChE (**c**), 1-NA (**d**), and 1-NB (**e**) under varying water conditions (well-watered vs. drought) and aphid infestation status (with/without peach aphids). Results are presented as mean ± SD of three replicates. Different lowercase letters indicate means that are significantly different according to the LSD test (*p* < 0.05). Drought + FL, Drought + TR, No Drought + FL, and No Drought + TR (FL = flonicamid; TR = thiacloprid). (glutathione-S-transferase (GST), acetylcholinesterase (AChE), butyrylcholinesterase (BuChE), 1-naphthyl acetate (1-NA), and 1-naphthyl butyrate (1-NB)).

The study investigated the association between aphid resistance attributes and pesticide efficacy under drought stress by analyzing GST, AChE, BuChE, 1-NA, and 1-NB activities. The study showed a significant insecticide × drought interaction effects on the all-enzyme activities (*p* < 0.01). Generally, enzyme activities were elevated under drought stress conditions (Figure 3a–e). Insecticides application had a significant effect on GST (df = 2, F = 7069.874, *p* < 0.01), AChE (df = 2, F = 1745.918, *p* < 0.01), BuChE (df = 2, F = 6863.265, *p* < 0.01), 1-NA (df = 2, F = 8087.456, *p* < 0.01), and 1-NB (df = 2, F = 120,608.112, *p* < 0.01) activities. Moreover, drought condition significantly affected aphid GST (df = 1, F = 5671.356, *p* < 0.01), AChE (df = 1, F = 99.735, *p* < 0.01), BuChE (df = 1, F = 884.405, *p* < 0.01), 1-NA (df = 1, F = 681.801, *p* < 0.01), and 1-NB (df = 1, F = 75,440.662, *p* < 0.01) activities. Notably, GST activities were highest in aphids on flonicamid-treated plants under both drought (2.5 ± 0.06) and well-watered (2.1 ± 0.1) conditions and least on thiacloprid treatments (1.833 ± 0.05 and 1.5 ± 0.05, respectively) (Figure 3a). Aphid AChE content under drought (259.2 ± 1.86) and those under well-watered (208.1 ± 2.53) were highest on flonicamid treatments and least on thiacloprid treatments (190.5 ± 11.78 and 176.5 ± 5.97, respectively) (Figure 3b). Moreover, BuChE activity was highest in aphids on flonicamid-treated plants under both drought (261.3 ± 2.65) and well-watered conditions (212.3 ± 2.68), and lowest on thiacloprid-treated plants (198.1 ± 1.90 and 143.1 ± 3.70, respectively) (Figure 3c). Similarly, 1-NA activity was highest on flonicamid treatments under both drought (205.5 ± 3.17) and well-watered (184.8 ± 2.74) conditions and least on thiacloprid treatments (170.1 ± 1.90 and 144.3 ± 1.49, respectively) (Figure 3d). Aphid 1-NB activity was also highest on flonicamid treatments under both drought (151.1 ± 2.21) and well-watered (121.1 ± 2.21) conditions and least on thiacloprid treatments (101.6 ± 2.21 and 62.6 ± 2.21, respectively) (Figure 3e). Overall, flonicamid-treated aphids exhibited the highest GST, AChE, BuChE, 1-NA, and 1-NB activities, regardless of drought conditions (Figure 3a–d).

### 2.4. Host Plant Physiological Response to Drought and Aphid Treatments

**Figure 4:**
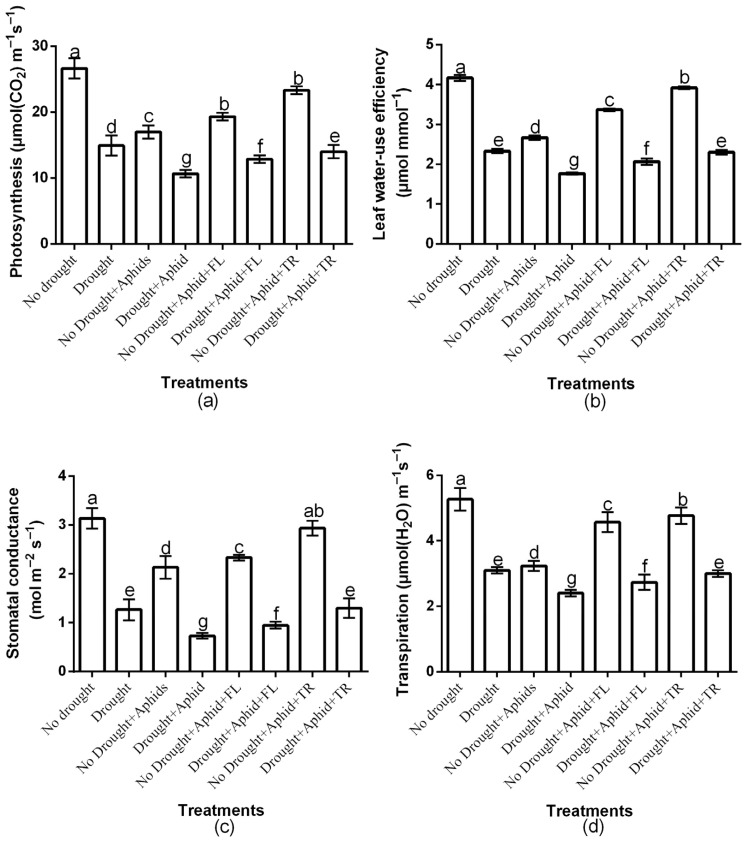
Effects of insecticides on key plant physiological traits: photosynthesis (**a**), leaf water use efficiency (**b**), stomatal conductance (**c**), and transpiration (**d**), under well-watered and drought conditions, with and without peach aphid infestation. Data are presented as mean ± SD of three replicates. Different lowercase letters indicate means that are significantly different according to the LSD test (*p* < 0.05). Combination of insecticide treatments involved Drought + FL, Drought + TR, No Drought + FL, and No Drought + TR (FL = flonicamid; TR = thiacloprid).

The study showed a significant (*p* < 0.01) insecticides × drought × aphid interaction effect on net photosynthesis, water-use efficiency, stomatal conductance, and transpiration rate (Figure 4a–d). Moreover, there was a significant insecticide × drought interaction effects on net photosynthesis (df = 2, F = 22.167, *p* < 0.01), water-use efficiency (df = 2, F = 13.142, *p* < 0.01), stomatal conductance (df = 2, F = 14.208, *p* < 0.01), and transpiration rate (df = 2, F = 15.391, *p* < 0.01). There was also a significant drought × aphids interaction effects on net photosynthesis (df = 1, F = 278.876, *p* < 0.01), water-use efficiency (df = 1, F = 39.351, *p* < 0.01), stomatal conductance (df = 1, F = 242.808, *p* < 0.01), and transpiration rate (df = 1, F = 43.103, *p* < 0.01). However, the two-way interactions effects of insecticides and aphids was not significant (*p* > 0.05). Drought and aphid treatments significantly (*p* < 0.01) reduced these parameters compared to the control. Notably, Thiacloprid-treated plants showed higher photosynthesis under both well-watered (23.3 ± 0.57) and drought conditions (14 ± 1) compared to flonicamid-treated plants (19.3 ± 0.57 and 12.8 ± 0.57, respectively) (Figure 4a). Thiacloprid-treated plants also showed higher water-use efficiency under both well-watered (3.92 ± 0.03) and drought conditions (2.3 ± 0.05) compared to flonicamid-treated plants (3.3 ± 0.03 and 2.06 ± 0.07, respectively) (Figure 4b). Moreover, thiacloprid-treated plants exhibited higher stomatal conductance under both drought (1.3 ± 0.2) and well-watered conditions (2.9 ± 0.15) compared to flonicamid-treated plants (0.95 ± 0.07 and 2.3 ± 0.5, respectively) (Figure 4c). Similarly, transpiration rate was higher on thiacloprid-treated plants under both drought (3 ± 0.1) and well-watered conditions (4.7 ± 0.25) compared to flonicamid-treated plants (2.7 ± 0.23 and 4.5 ± 0.30, respectively) (Figure 4d). Overall, thiacloprid-treated plants showed superior leaf physiological performance, with higher net photosynthesis, water-use efficiency, stomatal conductance, and transpiration rate under both water conditions (Figure 4a–d).

### 2.5. Host Plant Biomass Response to Drought Stress and Aphid Treatments

**Figure 5:**
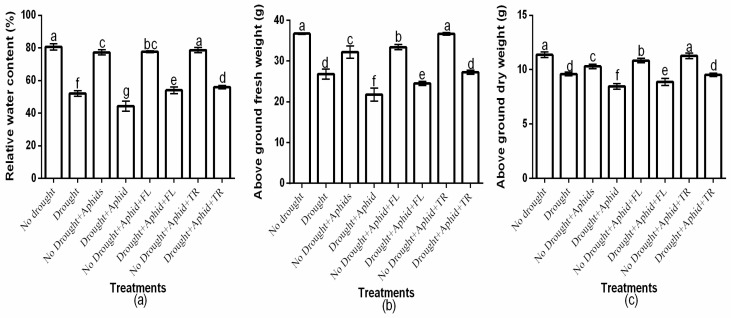
Effects of insecticides on plant growth and water relations: relative water content (**a**), aboveground fresh weight (**b**), and aboveground dry weight (**c**), under varying water conditions (well-watered vs. drought) and peach aphid infestation (with/without). Data are presented as mean ± SD of three replicates. Different lowercase letters indicate means that are significantly different according to the LSD test (*p* < 0.05). Drought + FL, Drought + TR, No Drought + FL, and No Drought + TR (FL = flonicamid; TR = thiacloprid).

The study showed a significant (*p* < 0.01) insecticides × drought × aphid interaction on host plant relative water content, above-ground fresh weight, and above-ground dry weight (Figure 5a–c). Moreover, there was a significant insecticide × drought interaction effects on relative water content (df = 2, F = 27.225, *p* < 0.01), above-ground fresh weight (df = 2, F = 505.218, *p* < 0.01), and above-ground dry weight (df = 2, F = 252.278, *p* < 0.01). There was also a significant drought × aphids interaction effects on relative water content (df = 1, F = 14.617, *p* < 0.01), above-ground fresh weight (df = 1, F = 72.837, *p* < 0.01), and above-ground dry weight (df = 1, F = 210.162, *p* < 0.01). However, the two-way interactions effects of insecticides and aphids was not significant (*p* > 0.05). Well-watered treatments had significantly (*p* < 0.01) higher values for these parameters compared to drought treatments. Drought and aphid infestation significantly (*p* < 0.01) reduced relative water content, above-ground fresh weight, and above-ground dry weight (Figure 5a–c). Notably, thiacloprid-treated plants exhibited the highest relative water content under both well-watered (78.7% ± 1.52) and drought conditions (56% ± 1), whereas flonicamid-treated plants had the lowest values (77.7% ± 0.57 and 54% ± 2, respectively) (Figure 5a). Thiacloprid-treated plants exhibited higher above-ground fresh weight under both well-watered (36.7 ± 0.32) and drought conditions (27.3 ± 0.47) compared to flonicamid-treated plants (33.4 ± 0.62 and 24.5 ± 0.5, respectively) (Figure 5b). Similarly, above-ground dry weight was greater on thiacloprid-treated plants under both well-watered (11.3 ± 0.25) and drought conditions (9.5 ± 0.15) compared to flonicamid-treated plants (10.8 ± 0.20 and 8.9 ± 0.32, respectively) (Figure 5c). Overall, thiacloprid-treated plants showed superior growth performance, with higher above-ground fresh and dry weights under both water conditions (Figure 5a–c).

## 3. Discussion

Drought-induced changes in plant physiology, particularly turgor pressure and sap composition, affect nutrient availability and quality [20], resulting in varying aphid performance on water-stressed plants. Drought has been found to have a positive [21], negative [22,23], or neutral [24] effect on aphid performance. In the current study, aphids performed better on drought-treated plants, demonstrating higher host acceptance, survival rate, colonization success, and average daily reproduction compared to those on well-watered plants. This finding is consistent with the previous research, which found that aphid performance on plants increased during drought stress [21]. Other studies, on the other hand, have found that aphids perform better on well-watered plants [5,25], emphasizing the need for more research into drought-induced physiological changes in plants and their impact on phytophagous insects.

*Myzus persicae*, a well-known aphid species, prefers leaves with high nutrient concentrations, especially immature and older leaves of Brassicaceae plants [26]. Notably, elevated levels of essential amino acids in the phloem sap of *Solanum tuberosum* L. have been shown to improve *M. persicae* survival, population growth rate, and fecundity while shortening development time [27]. Moreover, Khan et al. [21] found that water stress in cabbage (*Brassica oleracea* L. var. italic) increased sugar levels and decreased glucosinolate concentrations, leading to increased aphid populations. This is an indication that the drought condition probably increased the synthesis and accumulation of primary metabolites which served as source of nutrition in the host plants for enhanced aphid’s performance.

This finding is consistent with the “plant stress hypothesis,” which proposes that host plant stress can increase the availability of nitrogenous compounds, thereby enhancing herbivorous insect reproduction and survival [28]. The hypothesis predicts improved insect performance due to increased primary metabolites in the phloem, but it also recognizes that excessive stress can result in decreased performance due to increased osmoregulation demands. Plant stress increases its value as a food source for insects, which then decreases as the plant ages. Aphids most likely rely on water content for survival and primary metabolites for reproduction. Thiacloprid exhibited a greater lethal effect on green peach aphids, significantly impairing host acceptance, survival, colonization efficacy, and average daily reproductive output, irrespective of drought conditions. Notably, thiacloprid-treated plants demonstrated enhanced above-ground biomass production, characterized by increased fresh and dry weights, under both well-watered and drought regimes. The aphid tolerance index, which measures stress resistance, also revealed that green peach aphids were more sensitive to thiacloprid than flonicamid. Interestingly, plants treated with flonicamid had higher aphid water content but lower relative water content, indicating that aphids were more resistant to flonicamid treatments than thiacloprid.

The impact of drought stress on plant metabolism and aphid response is a complex phenomenon that requires further exploration. Our findings suggest that changes in plant metabolism under drought stress may have significant implications for pesticide efficacy. Drought stress could significantly impact plant metabolites, which may possibly influence aphid resistance to pesticides. Previous study showed that drought stress alters the production of primary metabolites, such as sugars, amino acids, and organic acids, essential for plant growth and development [27]. Also, drought stress induces the production of secondary metabolites, such as phenolics, terpenes, and alkaloids, which play a crucial role in plant defence against insect pests [22]. These changes in plant chemistry due to drought stress can alter the nutritional quality and toxicity of the plant to aphids, potentially affecting their susceptibility to pesticides. Aphids may adapt to these changes by modifying their own metabolic pathways, which could influence their resistance to pesticides. Consequently, the altered plant chemistry and aphid adaptation can impact the efficacy of pesticides, potentially leading to reduced control of aphid populations.

In this research, aphids showed higher resistance to both pesticides under drought stress plants compared to those under drought-free plants. This indicates drought stress could induce plant mechanisms, which can potentially affect pesticide efficacy. Moreover, changes in plant metabolites induced by drought stress may enhance aphids detoxification mechanisms to counteract the effects of pesticides. This is particularly relevant for flonicamid, a selective aphicide that relies on specific plant-aphid interactions to exert its toxic effects. In the context of Integrated Pest Management (IPM), breeding crops with improved drought tolerance can reduce the impact of water stress on plant–aphid interactions, while implementing cultural controls, such as irrigation management, can also minimize the impact of water stress on plant-aphid interactions. Also, rotating pesticides, including flonicamid, can help manage resistance development in aphid populations. We therefore speculate that the impact of drought stress on plant metabolites can significantly influence aphid resistance to pesticides, highlighting the complex interactions between plants, herbivores, and environmental stressors. Therefore, understanding the impact of drought stress on plant metabolites and aphid resistance to pesticides is crucial for developing effective integrated pest management strategies.

Insect resistance to pesticides is primarily driven by the activity of detoxifying enzymes, which improve insects’ metabolic capacity to counteract toxic compounds. The efficacy of insect detoxification is reflected in the responsiveness of detoxifying enzymes to insecticides. In insects, carboxylesterase (CarE), glutathione-S-transferase (GST), acetylcholinesterase (AChE), and cytochrome P450 (CYP450) are pivotal enzymes that mediate metabolic resistance to a broad spectrum of pesticides. Notably, exposure to various insecticides can modulate the activity of these enzymes, either through induction or inhibition, thereby influencing the evolution of insecticide resistance. Research has shown that increased detoxifying enzyme activity is associated with higher resistance ratios in thrips strains. Generally, GST, AChE, BuChE, 1-NA, and 1-NB activities were higher under drought stress, compared to the well-watered conditions. Insects possess a dynamic detoxification system, wherein enzymes are activated by diverse external and internal stimuli, enabling rapid adaptation to environmental stressors, including insecticides [7], thermal extremes [8], and elevated carbon dioxide levels [9]. This suggests that the accumulation of detoxification enzymes in this experiment is perhaps due to the combined effect of the drought and insecticides on the aphids.

Comparatively, aphid GST, AChE, BuChE, 1-NA, and 1-NB contents were highest on flonicamid treatments under both drought and well-watered conditions and least on thiacloprid treatments. It appears that the increased activity of metabolic detoxification enzymes is likely to contribute to insecticide resistance. Research has demonstrated a positive correlation between detoxification enzyme activity and resistance to organophosphorus insecticides in *Nilaparvata lugens* [29]. Moreover, elevated detoxification enzyme activity in this species enhances metabolism of spirotetramat, resulting in increased resistance [30]. Similarly to these findings, the decreased susceptibility of green peach aphids to flonicamid could be attributed to the higher accumulation of detoxification enzyme activities, suggesting that GST, AChE, BuChE, 1-NA, and 1-NB activities contribute to the detoxification metabolism of flonicamid insecticide. Research findings have indicated that insecticides elicit distinct responses in insect defence enzyme systems, with changes in enzyme activity being associated with either insect mortality due to toxicity or the evolution of resistance [30]. Insects use defence mechanisms against insecticides, such as upregulating detoxifying enzymes like glutathione-S-transferase (GST), acetylcholinesterase (AChE), and carboxylesterase (CarE). Sub-lethal exposure to insecticides like imidacloprid, diflubenzuron, and abamectin has been shown in studies to increase CarE and GST activity in certain insect species [12]. Specifically, AChE has been noted to have the ability to confer resistance to carbamate and organophosphate insecticides [12].

We hypothesize that the modulation of acetylcholinesterase (AChE) activity is essential for the development of pesticide resistance in aphids, with drought stress potentially influencing this relationship. AChE is reported to regulate neurotransmission by hydrolyzing the neurotransmitter acetylcholine in the insect nervous system. *M. persicae* populations have developed resistance to insecticides by modifying specific AChE gene variants, such as the S431F mutation [31]. This suggests that *M. persicae* exhibited reduced sensitivity to flonicamid, likely due to drought stress upregulating AChE activity in the aphid, hence augmenting their pesticide resistance. While the precise mechanisms underlying this link between drought stress, AChE activity, and pesticide resistance in green peach aphid remain incompletely elucidated, research indicates that drought stress may trigger the expression of stress-responsive genes implicated in detoxification processes, hence contributing to pesticide resistance [32]. The relationship among drought stress, AChE activity, and pesticide resistance in green peach aphid is complex and influenced by numerous factors, such as plant physiology and aphid behaviour. Therefore, understanding this relationship is crucial for developing effective pest management approaches that account for environmental stressors like drought.

Furthermore, studies have shown that treatment with spinetoram induces significant increases in CarE and GST activity in *F. occidentalis*, while concurrently decreasing AChE activity [13]. Although, aphids’ changes in detoxification enzyme activities were generally upregulated, they were relatively lower under thiacloprid insecticides treatments. The increased activity of detoxifying enzymes in green peach aphids treated with thiacloprid indicates that the aphids have developed tolerance to the insecticide. However, the internal defence mechanism was not enough to protect them from the lethal effect of this chemical. This is an indication that green peach aphids are susceptible to thiacloprid insecticides under both water conditions, as the detoxifying enzymes may not have effectively neutralized the oxidative radicals produced by the thiacloprid insecticide. This was confirmed by demonstrating low aphids performance and higher leaf net photosynthesis, water-use efficiency, stomatal conductance, and transpiration rate under both water conditions.

It appears that aphids’ insecticides resistance mechanisms are enhanced by drought stress as a result of host plant physiological modifications. Noticeably, in this research, detoxification enzyme activities were higher in aphids under drought stress plants compared to those under drought-free plants that indicates that water deficit differences might allow aphids to develop physiological adaptations to increase their metabolic resistance to insecticides. Green peach aphids can adapt to drought and pesticide stress by modifying their physiology to increase their tolerance to insecticides. Therefore, insect resistance monitoring and management under drought conditions need to be considered in pests managements. While our results provide valuable insights into the impact of water stress on plant-aphid interactions, the small sample size may limit the statistical power and generalizability of our findings. Future studies with larger sample sizes would be necessary to confirm and expand upon our results. Despite these limitations, our study provides valuable insights into the complex interactions between water stress, pesticides effects, and aphid response. Understanding these interactions is crucial for developing effective Integrated Pest Management (IPM) strategies that take into account the dynamic interplay between plants, aphids, and pesticides.

## 4. Materials and Methods

### 4.1. Insect Collection and Culturing

Adults of *M. persicae* were collected from the fields in Jeonju, Republic of Korea (35.82930° N; 127.0409° E). The insect culture was established on cabbage plants in well-ventilated acrylic cages and maintained under controlled conditions, specifically a temperature regime of 25 ± 1 °C, relative humidity of 75 ± 10%, and a photoperiod of 16 h light and 8 h darkness.

### 4.2. Growth Conditions and Plant Material

Cabbage seeds from the Sakata Seed Company, Seoul, Republic of Korea, were planted individually in pots measuring 12.5 cm in diameter and 9.5 cm deep and filled with 2 kg loamy soil. The pots were placed in a controlled greenhouse environment at the National Institute of Agricultural Sciences in Jeonju, Republic of Korea, under specific conditions: temperatures ranging from 25 to 35 °C during the day and 18–22 °C at night, humidity levels between 45 and 55%, and light intensity of 15,000–18,000 lux.

### 4.3. Experimental Design and Treatments

The experiment involved two drought levels (stressed and well-watered), two aphid infestation levels (infested and non-infested), and three levels of pesticide treatment (flonicamid, FL (Ulala DF^®^, Ishihara Sangyo Kaisha, Ltd., Osaka, Japan), thiacloprid, (Albarin^®^, Bayer CropScience, Thane, Maharashtra, India), and a control) arranged in a randomized complete bloke design with three replications. One hundred and eight pots were used, with three pots per unit. Plants in the well-watered treatments were watered regularly to maintain optimal soil moisture, while drought-stressed plants had watering withheld 30 days after planting, reducing soil moisture to 40% of field capacity. Soil moisture levels were monitored and adjusted daily as needed. Daily measurements using a Delta-T Theta Probe (Delta-T Devices, Cambridge, UK) ensured consistent moisture levels across treatments.

### 4.4. Application of Insecticides

The insecticides application was performed according to the method described by Quandahor et al. [6]. Briefly, the two insecticides, thiacloprid (Albarin^®^, Bayer CropScience, Thane, Maharashtra, India, 0.024% concentration, 24 SC formulation) and Flonicamid (Ulala DF^®^, Ishihara Sangyo Kaisha, Ltd., Osaka, Japan, 0.1% concentration, 50 WG formulation) were sprayed using an atomized hand sprayer. Treatment application was performed on a 7 d interval.

### 4.5. Determination of Aphid Performance

This study assessed aphid performance under two drought conditions by infesting three shoots per treatment with 20 aphid nymphs (n = 20 per treatment). The assessment concentrated on evaluating host acceptance, survival rate, colonization success, and average daily reproduction. Host acceptance was quantified by enumerating the number of nymphs remaining on the plants after a three-day period. The survival rate was computed as the percentage of nymphs that matured into reproductive adults by day 16. Colonization success was evaluated using a weighted formula that combined adult and nymph counts at day 20 [5]. Average daily reproduction was calculated by dividing the total number of nymphs produced by the duration (20 d) of the infestation period.

### 4.6. Aphid Water Content (AWC) and Aphid Tolerance Index (ATI)

Aphid water content was determined as described by Guo et al. [19]. Briefly, adult aphids were collected from each plant 20 d after infestation. The fresh weight (FW) was measured, then they were dried at 60 °C for 24 h, and then weighed again to obtain the dry weight (DW). Aphid water content was calculated as the percentage of water loss using the formula:

(1) $$AWC=\frac{FW-DW}{DW}\times 100$$

Aphid tolerance index (ATI), which indicates the resilience of the aphids to pesticides, was calculated according to Wilkins [20] as follows:

(2) $$ATI=\frac{Aphids\;reared\;on\;treated\;plants}{Aphids\;reared\;on\;control\;plants}\times 100$$

### 4.7. Determination of Glutathione S-transferase (GST) Activity in Green Peach Aphids

Glutathione S-transferase (GST) activity was determined according to the method described by Chen et al. [33]. Green peach aphids (30 frozen adults per treatment) were homogenized in a 1 mL of 0.1 M phosphate buffer (pH 7.0) at 4 °C and centrifuged at high speed (10,000× *g*) for 10–15 min to obtain a supernatant containing the GST enzyme. The GST assay was performed by preparing a reaction mixture consisting of phosphate buffer (pH 7.0), reduced glutathione (GSH), and 1-chloro-2,4-dinitrobenzene (CDNB). The aphid supernatant was added to the reaction mixture, and the change in absorbance was measured at 340 nm over time using a microplate reader. GST activity was calculated based on the rate of CDNB conjugation and was expressed as mol/min/mg protein. Protein concentration in the aphid supernatant was determined using the Bradford method with bovine serum albumin (BSA) as the standard. Specific GST activity was calculated by dividing enzyme activity by protein concentration [34].

### 4.8. Determination of Acetylcholinesterase (AChE) Activity in Green Peach Aphid

The AChE activity was determined according to the method described by Chen et al. [33] The assay involved homogenizing 30 frozen adults per treatment in 1 mL of 0.1 M phosphate buffer (pH 7.0) at 4 °C, followed by centrifugation to obtain the enzyme extract. The enzyme extract was then mixed with acetylthiocholine iodide (ATChI) as the substrate and 5,5′-Dithiobis(2-nitrobenzoic acid) (DTNB) as the chromogenic reagent. The reaction mixture was incubated at 25 °C for 10–30 min. The absorbance was measured at 412 nm using a microplate reader. AChE activity was calculated using the formula: AChE activity (mol/min/mg protein) = (ΔA412/min)/(ε × protein concentration), where ε is the molar extinction coefficient of the DTNB-thiol complex. To determine the IC50 value, a range of inhibitor concentrations (0.1–100 μM) was used. The AChE activity was detected using a colorimetric method, where the thiocholine produced by AChE hydrolysis of ATChI reacted with DTNB to form a yellow-coloured 5-thio-2-nitrobenzoic acid (TNB) anion.

### 4.9. Determination of Butyrylcholinesterase (BuChE) Activity in Green Peach Aphid

Butyrylcholinesterase (BuChE) activity was determined according to the method described by Sun et al. [35]. Green peach aphids (30 frozen adults per treatment) were homogenized in 1 mL of 0.1 M phosphate buffer (pH 7.0) at 4 °C and centrifuged at high speed (10,000× *g*) for 10–15 min to obtain a supernatant containing the BuChE enzyme. The BuChE assay was performed by preparing a reaction mixture consisting of DTNB (5,5′-dithiobis-2-nitrobenzoic acid), and BTChI (butyrylthiocholine iodide). The aphid supernatant was added to the reaction mixture, and the change in absorbance was measured at 412 nm over time using a microplate reader. BuChE activity was calculated based on the rate of thiocholine formation and was expressed as mol/min/mg protein. Protein concentration in the aphid supernatant was determined using the Bradford method with bovine serum albumin (BSA) as the standard [34]. Specific BuChE activity was calculated by dividing enzyme activity by protein concentration.

### 4.10. Determination of 1-Naphthyl Acetate (1-NA) Activity in Green Peach Aphid

1-Naphthyl Acetate activity was determined according to the method described by Sun et al. [35]. Green peach aphids (30 frozen adults per treatment) were homogenized in a buffer (phosphate buffer, pH 7.0) and centrifuged at high speed (10,000× *g*) for 10–15 min to obtain a supernatant containing the 1-NA enzyme. The reaction mixture consisted of phosphate buffer, 1-NA, and the aphid supernatant. The reaction mixture was added to a 96-well microplate, and the formation of 1-naphthol was measured kinetically at 450–550 nm using a microplate reader after adding Fast Blue B salt. The activity was calculated based on the rate of 1-naphthol formation and was expressed as mol/min/mg protein. Protein concentration in the aphid supernatant was determined using the Bradford method with bovine serum albumin (BSA) as the standard [34]. Specific 1-NA activity was calculated by dividing enzyme activity by protein concentration.

### 4.11. Determination of 1-Naphthyl Butyrate (1-NB) Activity in Green Peach Aphid

1-Naphthyl Butyrate activity was determined according to the method described by Sun et al. [35]. Green peach aphids (30 frozen adults per treatment) were homogenized in a buffer (phosphate buffer, pH 7.0) and centrifuged at high speed (10,000× *g*) for 10–15 min to obtain a supernatant containing the 1-NB enzyme. The reaction mixture consisted of phosphate buffer, 1-NB, and the aphid supernatant. The reaction mixture was added to a 96-well microplate, and the formation of 1-naphthol was measured kinetically at 450–550 nm using a microplate reader after adding Fast Blue B salt. The activity was calculated based on the rate of 1-naphthol formation and was expressed as mol/min/mg protein. Protein concentration in the aphid supernatant was determined using the Bradford method with bovine serum albumin (BSA) as the standard [34]. Specific 1-NB activity was calculated by dividing enzyme activity by protein concentration.

### 4.12. Physiological Parameters

Physiological parameters were measured in three plants using two fully expanded leaves from each. The parameters assessed included net photosynthesis, stomatal conductance, transpiration rate, and water use efficiency. Measurements were taken using a LI-6400XT infrared gas analyzer (LI-COR Biosciences, Lincoln, NE, USA) with controlled conditions, including constant CO_2_ levels and artificial lighting, between 9:00 and 11:00 on healthy leaves.

### 4.13. Leaf Relative Water Content (RWC)

The method developed by Qunadahor et al. [5] was used to determine leaf relative water content (RWC). Four youngest fully expanded leaves were removed from three shoots and the fresh weight (FW) was determined immediately. The leaves were immersed in distilled water for 6 h, then removed, and the adhering water was blotted with tissue paper before weighing to obtain turgor weight (TW). The dry weight (DW) was measured after drying the leaves at 70 °C in an oven for 24 h. The relative water content (RWC) was calculated as follows:

(3) $$RWC=\frac{FW-DW}{TW-DW}\times 100\%$$

### 4.14. Measurement of Aboveground Biomass

Aboveground biomass was assessed following the method of Quandahor et al. [5]. Fresh shoot weight was measured immediately after harvest for fresh biomass, and then the shoots were oven-dried at 80 °C for 72 h to determine dry biomass.

### 4.15. Statistical Analysis

The data were analyzed using SPSS software (Version 19.0, SPSS Inc., Chicago, IL, USA) to determine the effects of water stress and aphid infestation on plant metabolism and aphid population dynamics. A two-way ANOVA model was used to analyze the aphid parameters, with water stress and pesticide treatments as the main factors. Whereas a three-way ANOVA model was used to analyze the host plant parameters, with water stress, aphid infestation and pesticide treatments as the main factors. Percentage data were transformed using the arcsine square root transformation to meet the assumptions of ANOVA. The treatment means were compared using LSD Test at a significance level of *p* < 0.05. The results are presented as means ± standard deviation (SD).

## 5. Conclusions

This study showed that thiacloprid treatments had a greater lethal effect on green peach aphid on well-watered plants or plants under drought conditions, as shown by decreased host acceptance, survival, colonization success, daily reproduction, and tolerance index. Green peach aphid, on the other hand, demonstrated tolerance to flonicamid treatments, as evidenced by increased host acceptance, survival, colonization success, daily reproduction, and tolerance index. Detoxification enzyme activities were generally increased in response to both insecticides, but to a lesser extent with thiacloprid treatments. We therefore speculate that GST, AChE, BuChE, 1-NA, and 1-NB enzyme activities played an important role in flonicamid detoxification, implying that green peach aphid may rapidly develop flonicamid resistance under drought conditions. Thiacloprid, on the other hand, has a high efficacy against green peach aphids, making it an excellent choice for areas where both aphid infestations and drought stress are a major concern.

## Data Availability

The original contributions presented in this study are included in the article. Further inquiries can be directed to the corresponding author.
